# Surface asymmetry induced turn-overed lifetime of acoustic phonons in monolayer MoSSe

**DOI:** 10.1016/j.isci.2023.106731

**Published:** 2023-04-28

**Authors:** Xuefei Yan, Xiangyue Cui, Bowen Wang, Hejin Yan, Yongqing Cai, Qingqing Ke

**Affiliations:** 1 School of Microelectronics Science and Technology, Sun Yat-Sen University, Zhuhai 519082, People’s Republic of China; 2 Guangdong Provincial Key Laboratory of Optoelectronic Information Processing Chips and Systems, Sun Yat-Sen University, Zhuhai 519082, People’s Republic of China; 3 Joint Key Laboratory of the Ministry of Education, Institute of Applied Physics and Materials Engineering, University of Macau, People’s Republic of China

**Keywords:** Acoustic property, Materials property, Physics

## Abstract

Recent successful growth of asymmetric transition metal dichalcogenides via accurate manipulation of different chalcogen atoms in top and bottom surfaces demonstrates exotic electronic and chemical properties in such Janus systems. Within the framework of density functional perturbation theory, anharmonic phonon properties of monolayer Janus MoSSe sheet are explored. By considering three-phonons scattering, out-of-plane flexural acoustic (ZA) mode tends to undergo a stronger phonon scattering than transverse acoustic (TA) mode and the longitudinal acoustic (LA) mode with phonon lifetime of ZA (1.0 ps) < LA (23.8 ps) < TA (25.8 ps). This is sharply different from the symmetric MoS_2_ where flexural ZA mode has the weakest anharmonicity and is least scattered. Moreover, utilizing non-equilibrium Green function method, ballistic thermal conductance at room temperature is found to be around 0.11 nWK^−1^nm^−2^, lower than that of MoS_2_. Our work highlights intriguing phononic properties of such MoSSe Janus layers associated with asymmetric surfaces.

## Highlights

• Flexural acoustic mode has a stronger scattering than other acoustic modes
• The ordering of lifetime of acoustic modes is opposite from that of MoS_2_
• The ballistic thermal conductance at 300 K is found to be 0.11 nWK^−1^nm^−2^

## Graphical abstract

**Figure:**
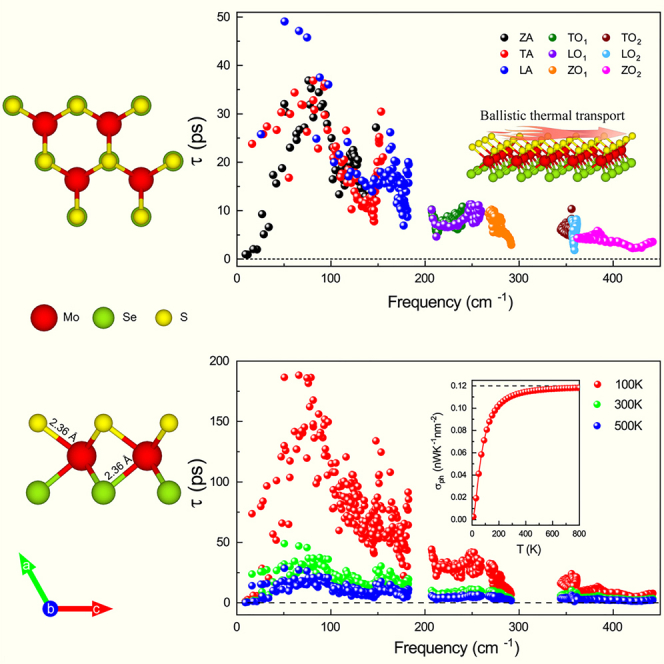

## Introduction

**Figure 1:**
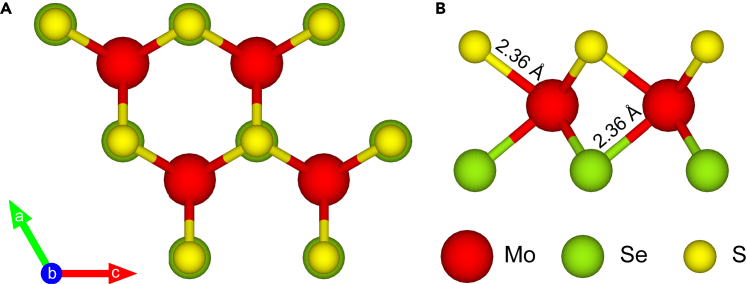
Schematic illustration of atomic structure (A and B) Top and side views of atomic structure of monolayer MoSSe.

In recent years, transition metal dichalcogenides (TMDs) have emerged as unique two-dimensional (2D) platforms for exploring quantum dynamics due to their unique electronic properties.^1^^,^^2^^,^^3^ As one of the typical monolayer TMDs, an atomically thin MoS_2_ monolayer is a direct-gap semiconductor with a strong spin-orbit coupling, which plays an important role in valleytronics^4^^,^^5^^,^^6^^,^^7^^,^^8^ and optical device applications.^9^ The excitations under electric field^7^^,^^8^^,^^10^^,^^11^ and dynamics of carriers of MoS_2_ including electrons/holes,^12^ excitons,^13^ as well as effects of the magnetic field^14^^,^^15^^,^^16^ and strain field^4^^,^^17^ were explored for potential nanoelectronics. Recently, by chemical vapor deposition, Janus MoSSe has been synthesized successfully.^18^^,^^19^ In contrast to mirror-symmetric MoS_2_, Janus MoSSe consisting of different chalcogen elements as the anionic sublayers is intrinsically asymmetric (seeing Figure 1A), which leads to broken symmetries and an intrinsic out-of-plane dipole moment. The effect is 2-folds: First, due to the lack of mirror symmetry, MoSSe monolayer is responsible for an incident angle-dependent second harmonic generation,^18^ out-of-plane piezoelectricity,^20^^,^^21^ as well as surface-enhanced Raman scattering of biomolecules.^22^ Second, the intrinsic out-of-plane dipole moment of monolayer MoSSe induces a potential gradient that facilitates water splitting,^23^ giant Rashba splitting,^21^^,^^24^ effective charge separation,^25^ extra long exciton lifetime,^26^ and remarkable photocatalytic activities.^27^^,^^28^^,^^29^

Except for the electronic properties, thermal properties of 2D materials are also of paramount importance in affecting the performance and reliability of nanodevices. Thus far, the thermal properties of some 2D materials, such as graphene,^30^^,^^31^^,^^32^^,^^33^ stanene,^33^^,^^34^^,^^35^ and monolayer TMDs,^33^^,^^36^^,^^37^^,^^38^^,^^39^^,^^40^^,^^41^^,^^42^ have been widely investigated. It has been reported that monolayer TMDs generally have a low thermal conductivity,^42^^,^^43^ which is beneficial for thermoelectric applications. Concerning Janus TMDs, Guo confirmed that the lattice thermal conductivity of monolayer MoSSe based on Boltzmann transport equation is 13.9 W/mK, which is between MoS_2_ and MoSe_2_.^44^ By performing equilibrium molecular dynamics simulations, Zhao et al. predicted that the lattice thermal conductivity of MoS_2(1−*x*)_Se_2*x*_ (*x* = 50%) alloy is 7.0 ± 1.8 W/mK, a 10-fold reduction compared with MoS_2_.^45^ More recently, Ding et al. reported that the minimum lattice thermal conductivity in bilayer MoSSe is about 1.57 W/mK.^46^ Thus, far most of the work focused on the lattice thermal conductivity in the diffusion regime. However, for atomically thin nanomaterials like MoSSe, the microscale and nanoscale thermal propagation could be ballistic. As far as we know, a quantitative estimation of ballistic thermal conductance of such asymmetric layers is still lacking. Most importantly, as a unique system with a regular mixture of S and Se from its parent MoS_2_ and MoSe_2_ compounds, MoSSe acts as an ideal platform for examining the phononic transmission and ballistic transport across such a half atomic-cleavage system.

In this paper, the thermal transmission of monolayer MoSSe is examined based on density functional perturbation theory and non-equilibrium Green function (NEGF) methods. Phonon dispersion and phonon lifetime (τ), phonon group velocity (*v*), as well as ballistic thermal conductance (*σ*_*ph*_) in per unit area are obtained. We derive that the ballistic thermal conductance per unit area of Janus MoSSe is 0.11 nWK^−1^nm^−2^ at room temperature, much lower than MoS_2_ and graphene. We also highlight the differences in phononic evolution and uncover the strikingly different behaviors of the lifetime order of acoustic modes between the Janus MoSSe and its predecessor MoS_2_.

## Results

### Phonons dispersion and phonon group velocities

In contrast to conventional TMDs, the rotation symmetry *C*_*2*_, mirror symmetry *σ*_*h*_, and improper rotation symmetry *S*_*3*_ in Janus monolayer MoSSe are broken because of different types of chalcogen atoms in the top and bottom surfaces of basal plane. As a consequence, Janus monolayer MoSSe has a lower symmetry *C*_*3v*_ instead of *D*_*3h*_ of conventional TMDs. Moreover, there are nine phonon modes (containing six optical phonon modes and three translational acoustic modes) at Γ point of Brillouin zone, since primitive cell of monolayer MoSSe contains three atoms. Associated with the lower *C*_*3v*_ symmetry of MoSSe, all the optical modes are infrared and Raman active. The phonon frequencies of these modes at the Γ point are calculated as 209.5 cm^−1^ (E^1^), 294.9 cm^−1^(${\text{A}}_{1}^{1}$), 361.1 cm^−1^ (E^2^), and 447.7 cm^−1^ (${\text{A}}_{1}^{2}$), and their vibrational patterns are shown in Figure S1. The results are in good agreement with experimental measurements.^47^^,^^48^

**Figure 2:**
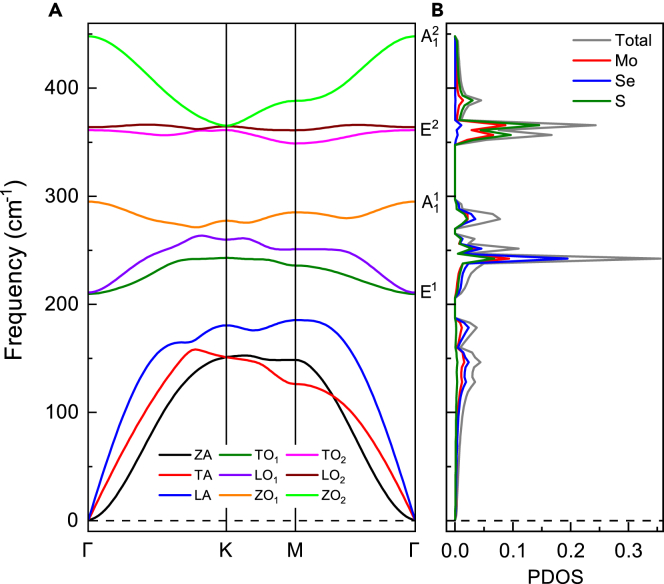
Crystal structure stability of monolayer MoSSe (A and B) Phonon dispersion and corresponding total and projected PDOS of monolayer MoSSe. No imaginary frequency in the whole Brillouin zones illustrates the thermal dynamic stability.

Phonon dispersions and phonon density of states (PDOS) of a monolayer MoSSe are depicted in Figure 2. Nine phonon branches exist and are labeled as transverse acoustic (TA), longitudinal acoustic (LA), flexural out-of-plane acoustic (ZA), transverse optical (TO_1_ and TO_2_), longitudinal optical (LO_1_ and LO_2_), as well as out-of-plane optical (ZO_1_ and ZO_2_) branches, respectively. The frequencies of all the phonon branches in the Brillouin zone are positive, which indicates that monolayer MoSSe can be structurally stable and generate restoring forces to resist atomic distortions. The acoustic and optical branches (LA and TO_1_) are well separated from each other. Comparing the corresponding PDOS in Figure 2B, one confirms that the phonon band gap between two optical branches (ZO_1_ and TO_2_) is around 50.4 cm^−1^, which is much larger than that between LA and TO_1_ branches (around 20 cm^−1^). Moreover, the low-frequency optical branches are mainly associated with the vibrations of Se atoms, whereas the high-frequency optical branches mainly involve the S atoms (see Figure 2B).

**Figure 3:**
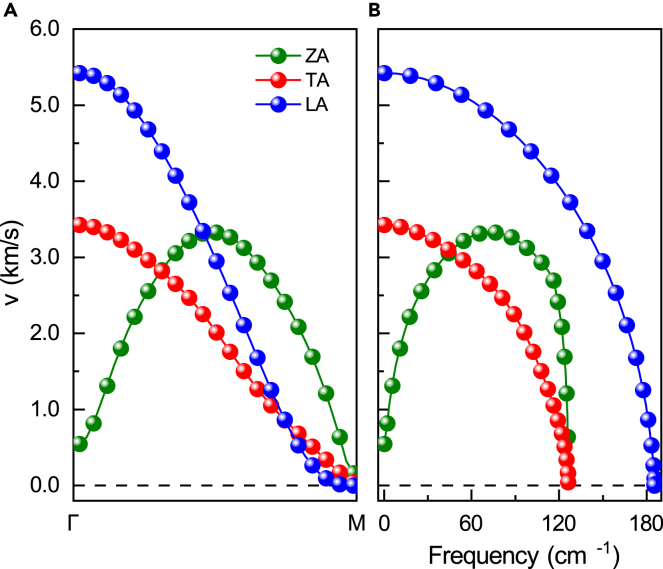
Phonon group velocities of acoustic phonon branches (A and B) Phonon group velocities of acoustic phonon branches with respect to wave vectors and with respect to frequencies.

Next, the phonon group velocities of acoustic phonon branches along with Γ-M high symmetrical line are calculated and shown in Figure 3A. Here, the phonon group velocity is defined as *v*_*n*_ *=* d*ω*_*n*_*/*d*q* with *n* being the *n*th phonon branch. The group velocity of the acoustic modes in the long-wavelength limit corresponds to the sound velocities at Γ point. For LA and TA branches, the sound velocities are respectively about 3.43 and 5.42 km/s, which are between MoS_2_ and MoSe_2_ monolayers (the details are compiled in Table S1).^36^^,^^44^^,^^49^ This difference is mainly related to mass differences between the constituent atoms (MoS_2_ < MoSSe < MoSe_2_). For the ZA branch, the momentum-dependent group velocity is zero at Γ and limited for long-wavelength phonons. The momentum-dependent group velocity curves follow a parabola-like curve peaked and bisected at the middle point of the Γ-M. Moreover, the group velocities for the LA, TA, and ZA branches as a function of frequency are shown in Figure 3B. The group velocities for the TA and the LA branches decline rapidly as the frequency increases, whereas that for the ZA branch first increases and then declines with the frequency. It is noted that the maximum sound velocity for the ZA branch in the middle of the Γ-M path is 3.33 km/s, which is quite close to the maximum velocity of the TA branch at the zone center.

### Phonon lifetime and ballistic thermal conductance

**Figure 4:**
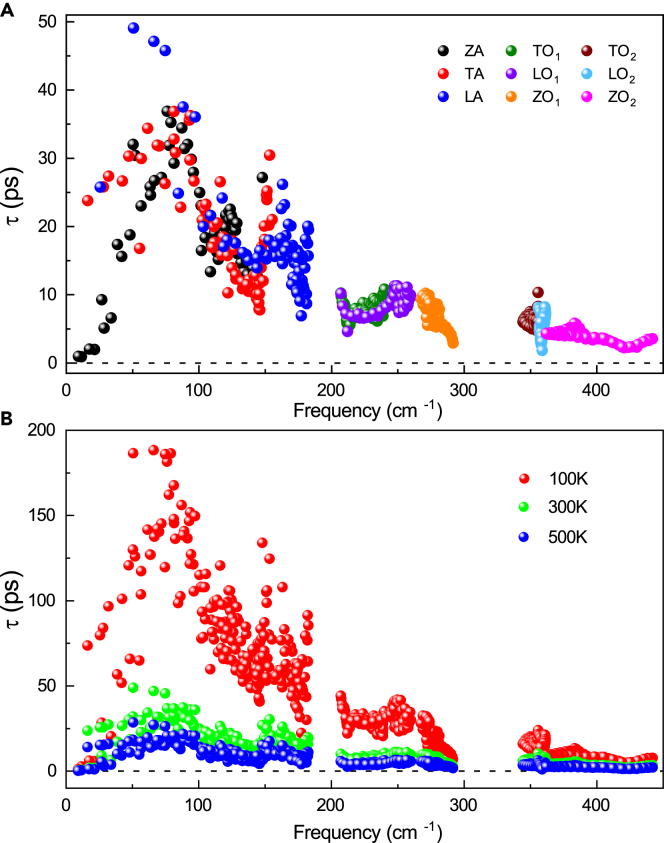
Phonon lifetime of all the phonon branches (A and B) Frequency-dependent phonon lifetime of all the phonon branches at room temperature and different temperatures.

The frequency-dependent phonon lifetime of phonon branches at room temperature (300 K) is derived and shown in Figure 4A, and the phonon lifetimes for all the phonon branches at Γ point are labeled in Table 1. It can be intuitively seen from Table 1 that the order of phonon lifetime for acoustic branches is ZA (1.0 ps) < LA (23.8 ps) < TA (25.8 ps), implying that the long-wavelength acoustic ZA branch undergoes a stronger phonon scattering than LA and TA branches. This is strongly different from the MoS_2_ case where the ZA mode around the zone center has the weakest anharmonicity [55] corresponding to the longest lifetime. Moreover, the dependence of phonon lifetime for LO_2_ branch with frequency is very sharp owing to the flat curve of dispersion with the frequency which is almost localized around 361.1 cm^−1^. The distribution of phonon lifetime is relatively spreading (Δτ ≈ 6.3 ps) while the phonons are overall short-lived (<10 ps). Such a short lifetime for the high-frequency optical modes means that phonon decay is quite fast. A short lifetime for the low-frequency optical modes is also observed at room temperature. However, the lifetime of low-frequency optical phonon modes increases up to 50 ps at 100 K. This increase is reasonable as the density of phonons becomes condensed and less activated by lowering the temperature, making those high-frequency modes less scattered (See Figure 4B). Similarly, with a reduction of temperature down to 100 K, the lifetime of acoustic modes is significantly prolonged. Such a huge contrast with temperature portends the phonon anharmonicity of MoSSe weakens as temperature decreases. This is consistent with the lattice thermal conductivity of MoSSe decreasing with increasing temperature.^44^

**Table 1:** Phonon frequencies and lifetime for three acoustic branches and four optical modes at the Γ point compared to experimental data

	ZA	TA	LA	E_1_	A1 1	E_2_	A2 1
*ω*/cm^−1^	0	0	0	209.5 212^a^ –	294.9 291^a^ 290^b^	361.1 357^a^ 354^b^	447.7 442^a^ 442^b^
*τ*/ps	1.0	23.8	25.8	10.2	2.9	8.3(8.2)	3.6

Note that phonon lifetime (*τ*) is displayed at the cutoff frequency (*ω*_*c*_) of 10 cm^−1^.

a Experimental data in ref.^47^.

b Experimental data in ref.^48^.

**Figure 5:**
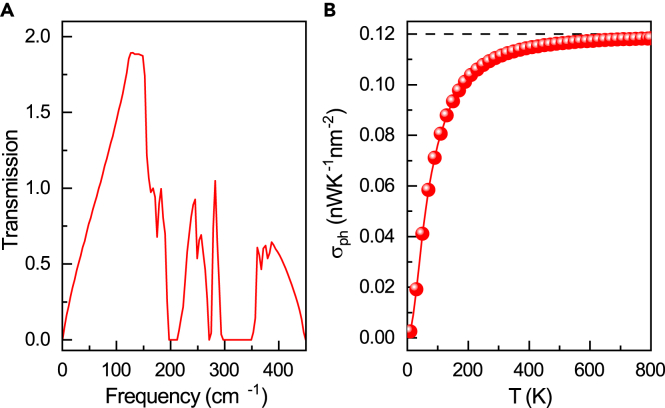
Thermal properties of monolayer MoSSe (A and B) Frequency-dependent phonon transmission and temperature-dependent ballistic thermal conductance of monolayer MoSSe were calculated by the NEFG method.

The issue of thermal conductance is critical for 2D nanodevices as they affect the performance and reliability of nanodevices. Here, we only consider phonon-related thermal conductance, since the Janus MoSSe shows the semiconducting behavior with a limited concentration of carriers like electrons and holes contributing to the thermal conduction. By adopting the NEGF method, the phonon transmission function and ballistic thermal conductance for a monolayer MoSSe sheet are calculated by Equation 1. Assuming the sample with a thickness of 2.1 Å, the cross-area of the sample amounts to 0.067 nm^2^. From Figure 5A , a transmission gap exists around the frequency of 200 cm^−1^, which corresponds to the forbidden band gap between acoustic and optical branches. Phonon transmission is significantly higher at low frequencies than at high frequencies, that is, the acoustic branches of monolayer MoSSe dominate the phonon thermal conductivity in ballistic thermal transport. This is also the key to the long lifetime of acoustic phonons (see Figure 4). The maximum phonon transmission for monolayer MoSSe is around 1.9, much smaller than that of monolayer MoS_2_.^36^ The result implies that the thermal conductance of monolayer MoSSe is lower than MoS_2_. As indicated in Figure 5B, the ballistic thermal conductance increases with the increase in temperature and levels off at around 600 K. The maximum ballistic thermal conductance per unit area is around 0.12 nWK^−1^ nm^−2^, around 40-fold smaller than that of graphene^50^ and comparable to that of monolayer stanene.^34^

## Discussion

In summary, Janus TMDs are unique systems appealing for activation of the multi-field coupling owing to intrinsic breaking of symmetry. Here, we have investigated phonon decay and ballistic phonon thermal transport of Janus monolayer MoSSe within a finite range of temperature. By extracting phonon dispersion, group velocity, phonon density of states, and phonon lifetime, we observe the following results: (a) we uncover the abnormal behavior of the strongest scatted ZA mode in this asymmetric Janus layer compared with the long-lived ZA mode in its symmetric predecessor MoS_2_. (b) We predict the LO_2_ branch is highly localized while its distribution of phonon lifetime (Δτ) is broad, acting as a possible decaying channel. (c) We predict the temperature evolution of phonon dynamics in MoSSe and explained the origin of the temperature-dependent variation of lattice thermal conductivity. By utilizing NEGF method, the ballistic thermal conductance of monolayer MoSSe at room temperature is found to be around 0.11 nWK^−1^nm^−2^, much smaller than other 2D materials such as graphene, MoS_2_, as well as black phosphorene. Our work sheds new light on the phononic behaviors related to the asymmetric Janus surface.

### Limitations of the study

In the current work, the differences in phononic evolution and transport of the Janus MoSSe and its host MoS_2_ and other 2D materials are estimated through examining the phonon lifetime and phonon thermal transport related to three-phonons Umklapp scattering and mass-difference scattering. However, higher-order phonon scattering process, such as four-phonon scattering, plays an important role in thermal properties of semiconductors even at room temperature. Particularly, in graphene, the relative contribution of the ZA phonon branch considering three-phonon scattering is significantly overestimated, more than twice that considering four-phonon scattering. Hence, the effect of four-phonon scattering is expected to also exist and alter the thermal anharmonicity of MoSSe.

## STAR★Methods

### Key resources table

**Table:** 

REAGENT or RESOURCE	SOURCE	IDENTIFIER
**Software and algorithms**		

Quantum Espresso	Giannozzi at el.^51^^,^^52^^,^^53^	https://www.quantum-espresso.org/
ShengBTE	Li at el.^61^	https://www.shengbte.org/home
Python3.7	Python Software Foundation	https://www.python.org/
ForTran95	ForTran Software Foundation	https://fortran-lang.org/en/

### Resource availability

#### Lead contact

Further information and requests for resources and reagents should be directed to and will be fulfilled by the lead contact, Yongqing Cai (yongqingcai@um.edu.mo).

#### Materials availability

This study did not generate new unique reagents.

### Method details

The interatomic force constants (IFCs) and phonon dispersion relations are calculated based on DFPT method. All the numerical calculations are performed by *Quantum ESPRESSO* code,^51^^,^^52^^,^^53^ with the local density approximation (LDA) as an exchange-correlation function. Norm-conserving fully relativistic pseudopotentials with plane-wave energy (charge density) cutoff of 105 Ry (1400 Ry) are used.^54^^,^^55^^,^^56^ The vacuum distance of the slab is greater than 15 Å. Brillouin zone integration is sampled with a 20 × 20 × 1 Monkhorst-Pack grid. Details of the convergence tests are provided in the supporting information (see Figures S2−S4). The energy difference and force convergence criteria are 10^–8^ eV and 10^–7^ eV/Å during the whole structural relaxation. After optimization, the equilibrium lattice constant of single-layer Mosse is 3.18 Å, which is less than the measured value of 3.25,^24^ since LDA usually underestimates the lattice constant. In the former DFPT calculation, the dynamical matrix is calculated by 9 × 9 × 1 ***q*** mesh.

In the ballistic transport regime, the ballistic thermal conductance per unit area can be given via the Landauer Büttiker formula

(Equation 1) $$\sigma_{\text{ph}}=k_{B}\int_{0}^{\infty }\frac{d\omega }{2\pi S}T_{\text{ph}}\left[\omega \right]\frac{{\left(\hbar \omega /k_{B}T\right)}^{2}e^{\hbar \omega /k_{B}T}}{{\left(e^{\hbar \omega /k_{B}T}-1\right)}^{2}}$$

where *k*_B_ and *T* denote Boltzmann constant and average temperature of the hot and cold baths, respectively. *ħ* refers to Plank constant and *T*_ph_[*ω*] refers to the phonon transmission function threading cross-area *S*. Here, the cross-area *S* can be defined as *S* = *a* × *W*, where *a* is the lattice constant of a transverse unit cell and *W =* 2.1 Å is the effective thickness of monolayer MoSSe. Furthermore, *T*_ph_[*ω*] in this paper is solved by the NEGF method.^34^^,^^36^^,^^57^^,^^58^ The *T*_ph_[*ω*] is obtained as

(Equation 2) $$T_{\text{ph}}\left[\omega \right]=\text{Tr}\left[G^{r}\Gamma_{L}G^{a}\Gamma_{R}\right]$$

where Γ_L_ (Γ_R_) refers to the coupling matrix between central region and leads, where *L (R)* subscript denotes left (right) lead. G^r^ (G^a^) is advanced (retarded) Green function. To calculate the Green function, the advanced Green function can be given by

(Equation 3) $$G^{r}={\left[\omega^{2}-K^{c}-\sum_{L}^{r}-\sum_{R}^{r}\right]}^{-1}$$

G^r^ = (G^a^)^†^ and *K*^*c*^ is the force constant matrix. $\sum_{L}^{r}$ ($\sum_{R}^{r}$) is a self-energy term, which is defined as

(Equation 4) $$\sum_{L}^{r}=H_{LC}^{†}g_{L}^{r}\left(\omega \right)H_{LC},\sum_{R}^{r}=H_{RC}^{†}g_{R}^{r}\left(\omega \right)H_{RC}$$

*H*_*L(R)C*_ represents coupling matrix between the lead *L (R)* and central region (C). $g_{L(R)}^{r}$ refers to a surface advanced Green function of *L (R)* lead. Hence, the coupling matrix Γ_L_ (Γ_R_) can be written as

(Equation 5) $$\Gamma_{L\left(R\right)}=i\left(\sum_{L}^{r}-\sum_{R}^{a}\right)$$

For real materials, the relaxation of phonons in resistive processes involve various scattering processes, for example, boundary scattering, defect scattering, three-phonons Umklapp scattering, and mass-difference scattering, among others. However, in the experiment, the scattering induced by boundary and defect can be suppressed via improving sample quality, thus behaving somehow extrinsically. Here, we only consider intrinsic scattering events including both three-phonons Umklapp scattering and mass-difference scattering. According to time-dependent perturbation theory^59^^,^^60^ and lifetime approximation, the phonon lifetime (*τ*) for phonon branch λ can be given as^33^

(Equation 6) $$\frac{1}{\tau }=\frac{1}{n_{\lambda }^{0}\left(n_{\lambda }^{0}+1\right)}\left(\sum_{\lambda^{\prime }\lambda^{\prime \prime }}^{+}\Gamma_{\lambda \lambda^{\prime }\lambda^{\prime \prime }}^{+}+\sum_{\lambda^{\prime }\lambda^{\prime \prime }}^{-}\frac{1}{2}\Gamma_{\lambda \lambda^{\prime }\lambda^{\prime \prime }}^{-}+\sum_{\lambda^{\prime }}\Gamma_{\lambda \lambda^{\prime }}\right)$$

where $n_{\lambda }^{0}$ is Bose distribution function and Γ_*λλ′*_ corresponds to mass-difference scattering. $\Gamma_{\lambda \lambda^{\prime }\lambda^{\prime \prime }}^{+}$ ($\Gamma_{\lambda \lambda^{\prime }\lambda^{\prime \prime }}^{-}$) is three phonon scattering processes. $\Gamma_{\lambda \lambda^{\prime }\lambda^{\prime \prime }}^{+}$ refers to the absorption processes in which the energy of only one phonon combines the energy of two incident phonons, whereas $\Gamma_{\lambda \lambda^{\prime }\lambda^{\prime \prime }}^{-}$ refers to the emission processes in which the energy of one incident phonon is dissipated via splitting into two phonons.

We employ the software package *ShengBTE* here to obtain numerical results for the phonon lifetime.^61^ Here, the third-order IFCs utilize a 4 × 4 × 1 supercell. To obtain the phonon lifetime accurately, a 27 × 27×1 ***q***-point grid is adopted to ensure convergence. To avoid divergence problems, the cutoff frequency (*ω*_*c*_) should be used in the formula a cutoff frequency.^30^^,^^62^ In previous studies on bulk graphene, Klemens et al. employed the frequency of acoustic branch ZO′ at the Γ point as *ω*_*c*_ (about 132 cm^-1^) according to the assumption of cross-surface coupling and heat transfer at this frequency.^62^ However, there are no acoustic branch ZO′ in monolayer MoS_2_. Cai et al. selected the 5% of the maximum frequency of the long-wavelength acoustic branch LA as the cutoff frequency (around 1.2 cm^-1^) in monolayer MoS_2_, grounded on assuming a constant sound velocity within the whole Brillouin zone and the umklapp scattering rates of phonons.^36^ Monolayer MoSSe, as a new Janus TMDs, has a similar structure to monolayer MoS_2_, and there is also no acoustic branch ZO'. Therefore, the cutoff frequency of 10 cm^-1^ in MoSSe, amounting to 0.5% of the frequency of the LA phonon at the M point (around 200 cm^−1^), is selected.

## Data Availability

• Any additional information required to reanalyze the data reported in this paper is available from the lead contact upon request. • This paper does not report original code. • All data reported in this paper will be shared by the lead contact upon request.
