# Supplementary material for: Surface asymmetry induced turn-overed lifetime of acoustic phonons in monolayer MoSSe
Source: iScience. 2023 Apr 28;26(5):106731. doi: 10.1016/j.isci.2023.106731 (PMC10197104; doi:10.1016/j.isci.2023.106731)
Supplement: Document S1. Figures S1–S4 and Table S1 [file mmc1.pdf]

iScience, Volume *26*

Supplemental information

Surface asymmetry induced turn-overed lifetime of acoustic phonons in monolayer MoSSe

Xuefei Yan, Xiangyue Cui, Bowen Wang, Hejin Yan, Yongqing Cai, and Qingqing Ke

Supplemental Information

Surface asymmetry induced turn-overed lifetime of acoustic phonons in monolayer MoSSe

Xuefei Yan [1, 2, 3], Xiangyue Cui [3], Bowen Wang [3], Hejin Yan [3],
Yongqing Cai [3, *], Qingqing Ke [1, 2, *]

[1] School of Microelectronics Science and Technology, Sun Yat-Sen University, Zhuhai 519082, People's Republic of China.

[2] Guangdong Provincial Key Laboratory of Optoelectronic Information Processing Chips and Systems, Sun Yat-Sen University, Zhuhai 519082, People's Republic of China

[3] Joint Key Laboratory of the Ministry of Education, Institute of Applied Physics and Materials Engineering, University of Macau, Taipa, Macau, People's Republic of China.

* Corresponding author: yongqingcai@um.edu.mo, keqingq@mail.sysu.edu.cn

Contents

Figure S1 The vibration figures of six high-frequency optical branches, where the blue arrows point out the atomic vibration directions. 1

Figure S2 The total energy of monolayer MoSSe as a function of Encut. 1

Figure S3 The total energy of monolayer MoSSe as a function of K-points. 2

Figure S4 The total energy of monolayer MoSSe as a function of Vaccuum Space. .2

Table S1 The group velocity of monolayer MoS_2, MoSSe, and $MoSe_2$ at Γ point for three acoustic modes along Γ-M direction, respectively. 3

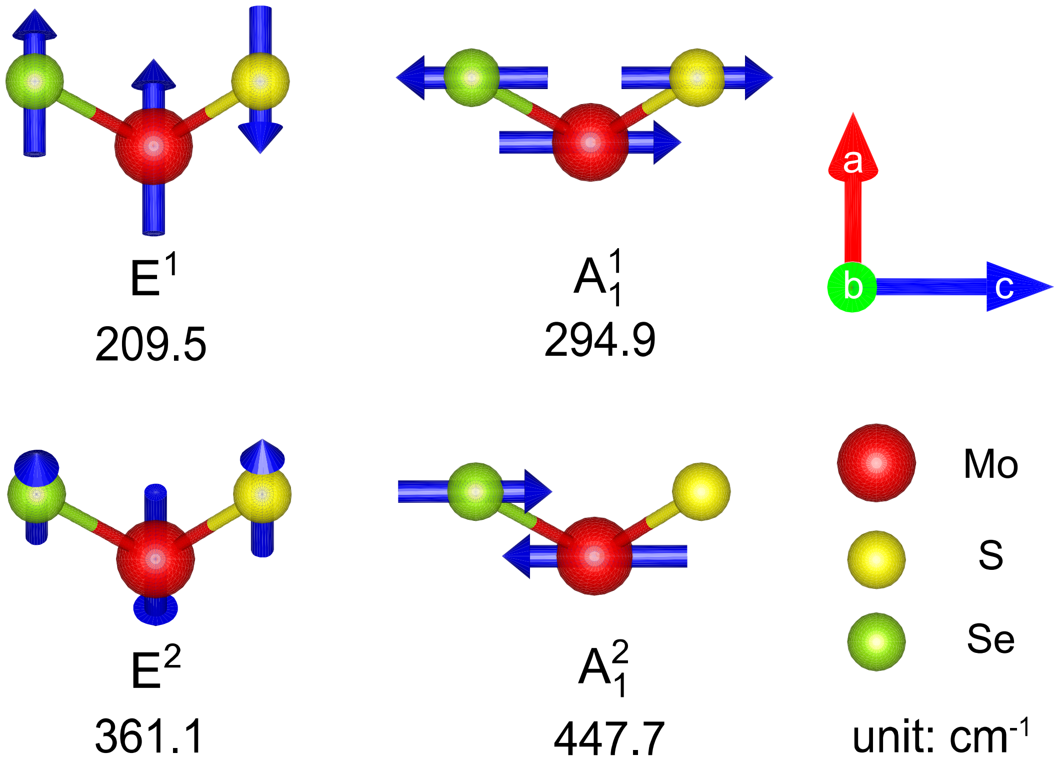

Figure S1 The vibration figures of six high-frequency optical branches, where the blue arrows point out the atomic vibration directions. Related to Figure 2A.

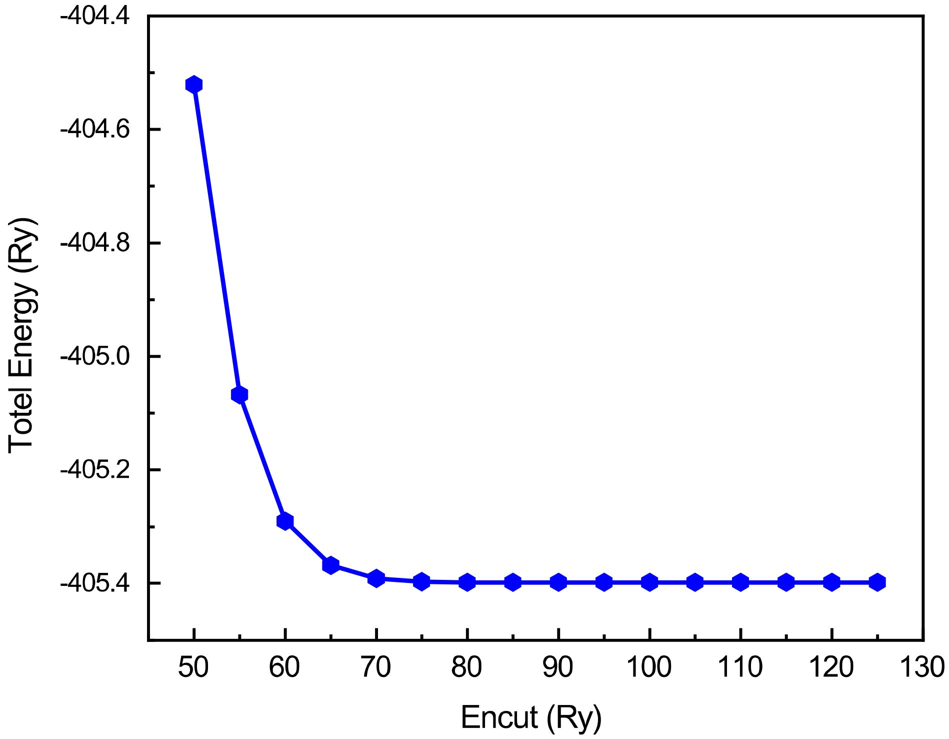

Figure S2 The total energy of monolayer MoSSe as a function of Encut. Related to STAR Methods.

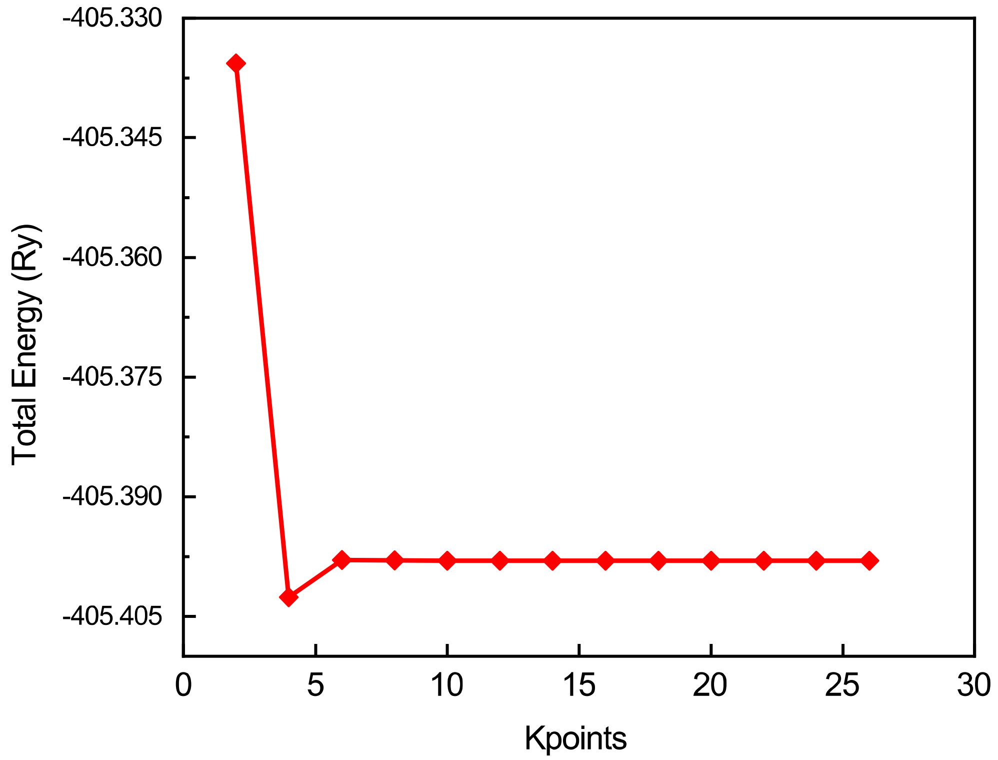

Figure S3 The total energy of monolayer MoSSe as a function of K-points. Related to STAR Methods.

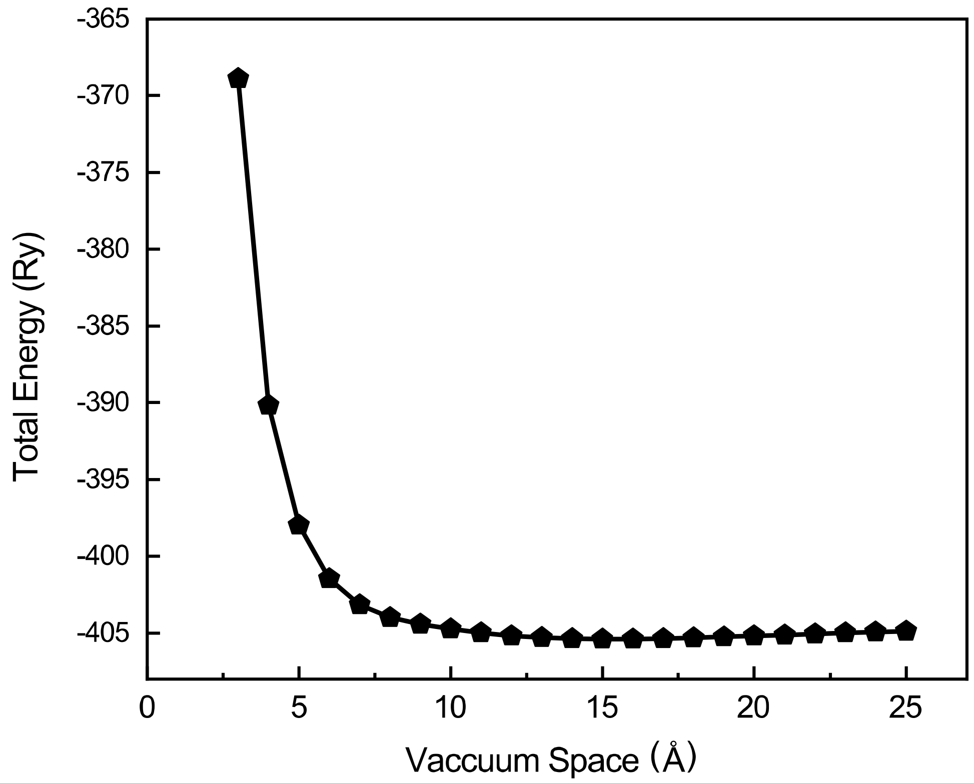

Figure S4 The total energy of monolayer MoSSe as a function of Vaccuum Space. Related to STAR Methods.

Table S1 The group velocity of monolayer MoS_2, MoSSe, and $MoSe_2$ at Γ point for three acoustic modes along Γ-M direction, respectively. Related to Figure 3A.

	v-TA (km/s)	*v*-LA (km/s)	Reference
MoS_2	4.36	6.96	Ref. [33]
	4.11	6.60	Ref. [38]
	4.09	6.55	Ref. [50]
MoSSe	3.43	5.42	This work
	3.55	5.62	Ref. [38]
$MoSe_2$	3.22	5.06	Ref. [38]
